# Supplementary material for: CoFee-L: A Model of Animal Displacement in Large Groups Combining Cohesion Maintenance, Feeding Area Search and Transient Leadership
Source: Animals (Basel). 2022 Sep 14;12(18):2412. doi: 10.3390/ani12182412 (PMC9495015; doi:10.3390/ani12182412)
Supplement: Supplementary file 1 [file animals-12-02412-s001.zip › animals-1865719-supplementary.pdf]

Supplementary Materials:

Video S1. A basic example with 10 particles to illustrate the 3 phases of CoFee-L. The green (resp. red) discs represent the active (resp. inactive) FAs, while the small black discs are the simulated individuals. The CM of the group and its displacement pattern are shown in red. The leaders are shown in orange.

Video S2. Example of a simulation of a day of monitoring, with a zoom on the area of interest in order to visualize the 140 particles as clearly as possible. The green (resp. red) discs represent the active (resp. inactive) FAs, while the small black discs are the simulated individuals. The CM of the group and its displacement pattern are shown in red. The leaders are shown in orange.

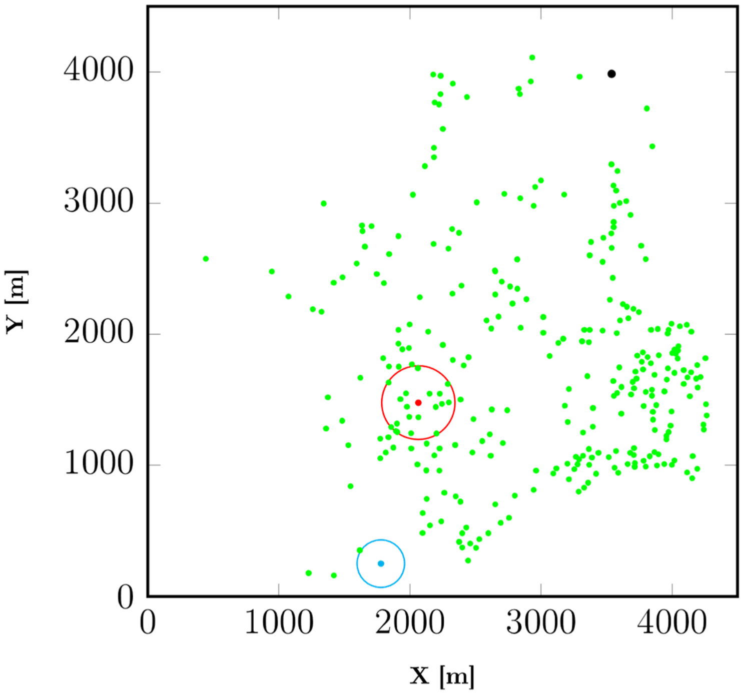

Figure S1. Map corresponding to the month of high abundance in the dry evergreen forest. Each green dot indicates a food source (FA > 0). The circles correspond to the detection range of 3 food sources for *abundanceReach* = 100, with different values of abundance (red: 2.8, cyan: 1.81, black: 0.27).

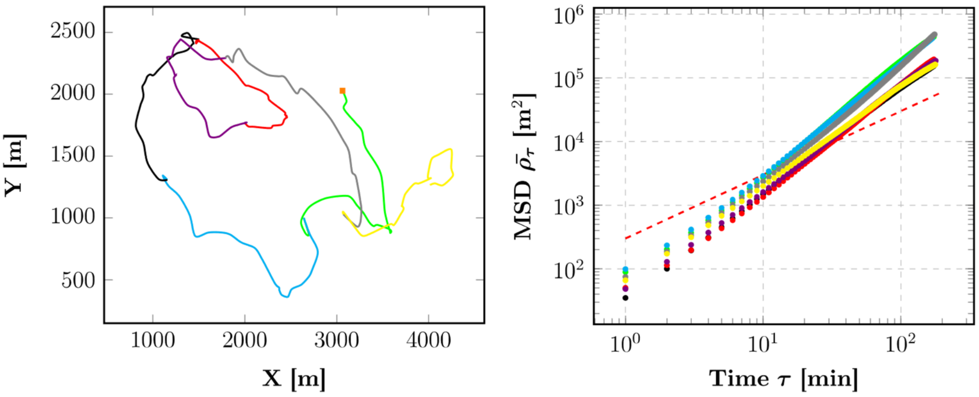

Figure S2. Evolution of the troop position (left), with the corresponding MSDs (right, mean α = 1.69 ± 0.08) for a tracking of 7 consecutive days during high food availability in plantations. The orange square indicates the departure of the troop and the dashed line represents a line of a unitary slope, i.e., for $\alpha = 1$.

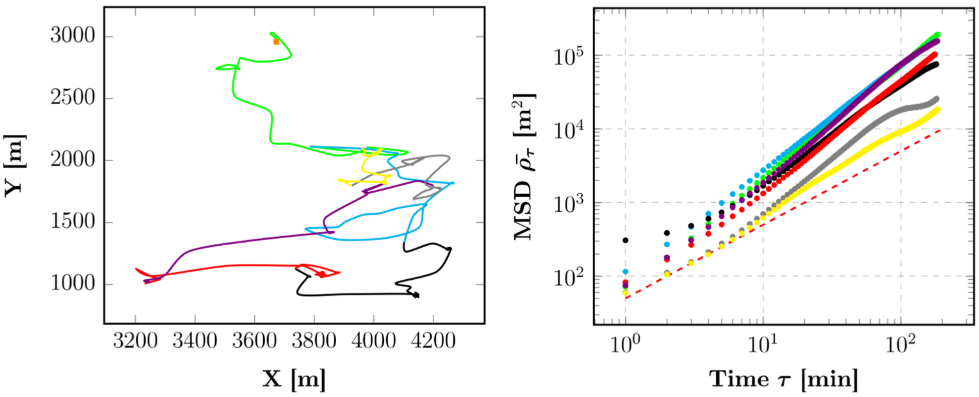

Figure S3. Evolution of the troop position (left), with the corresponding MSDs (right, mean α = 1.36 ± 0.15) for a tracking of 7 consecutive days during low food availability in dry evergreen forest and plantations. The orange square indicates the departure of the troop and the dashed line represents a line of a unitary slope, i.e., for $\alpha = 1$.

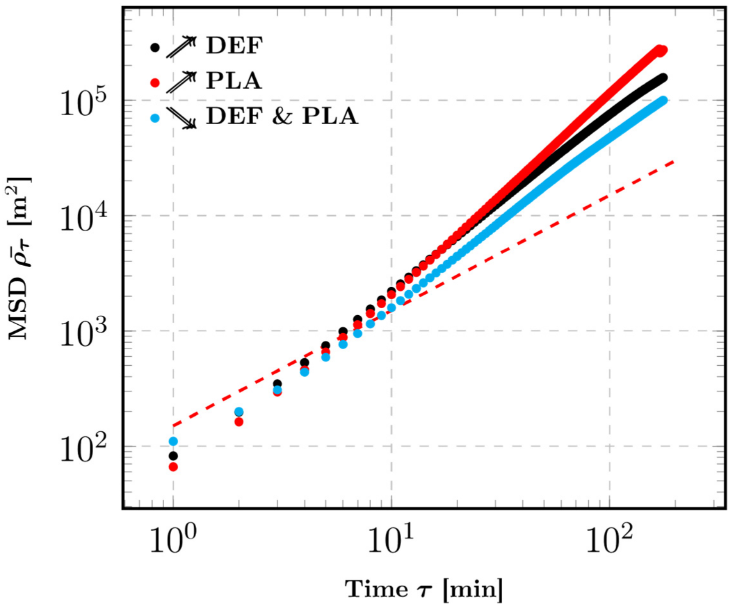

Figure S4. Average MSDs values of the troop for the three periods of fruit abundance. Each point represents the average of the 6 or 7 monitoring days, depending on the tracking conditions, for the period of observation considered. The dashed line represents a line of a unitary slope, i.e., for $\alpha = 1$.

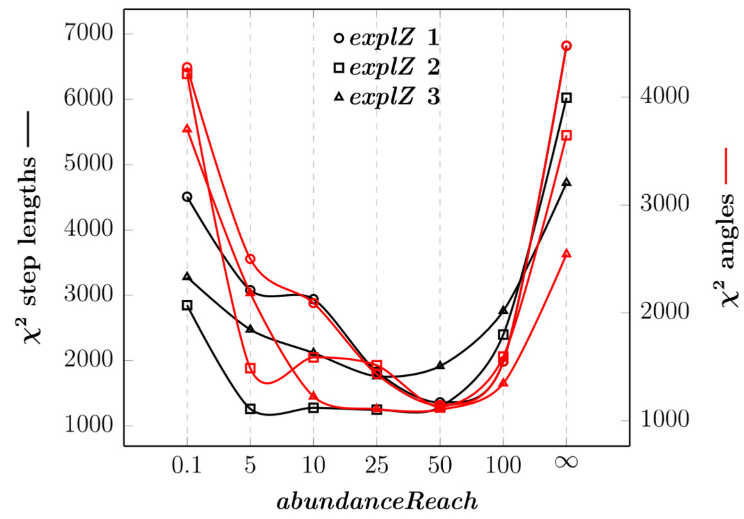

Figure S5. χ^2 for comparing step length and angle distributions between observed and simulated trajectories as a function of *abundanceReach*, for *velocity* = *levyRatio* = 1 and *explorationZone* = 1, 2 and 3. The black (resp. red) curves represent the χ^2 for step lengths (resp. angles) and are plotted along the left-hand (resp. right-hand) y-axis. For each simulation, the group of particles was simulated from the same starting point and on a map of high food availability in plantations.

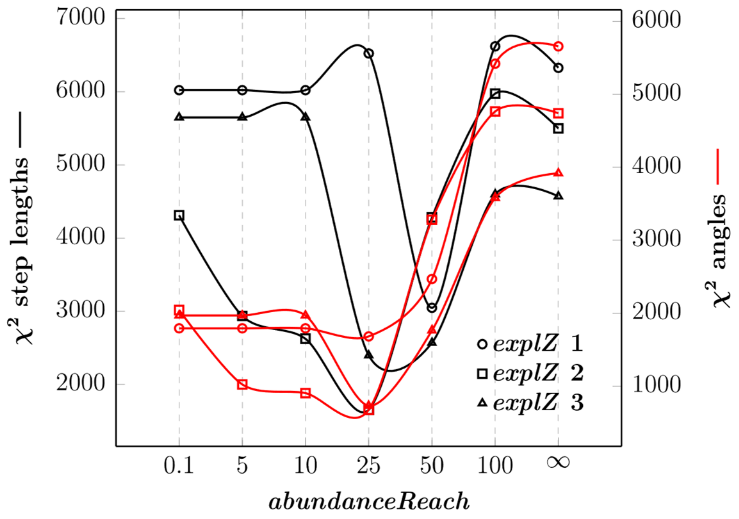

Figure S6. χ^2 for comparing step length and angle distributions between observed and simulated trajectories as a function of *abundanceReach*, for *velocity* = *levyRatio* = 1 and *explorationZone* = 1, 2 and 3. The black (resp. red) curves represent the χ^2 for step lengths (resp. angles) and are plotted along the left-hand (resp. right-hand) y-axis. For each simulation, the group of particles was simulated from the same starting point and on a map of low food availability in dry evergreen forest and plantations.

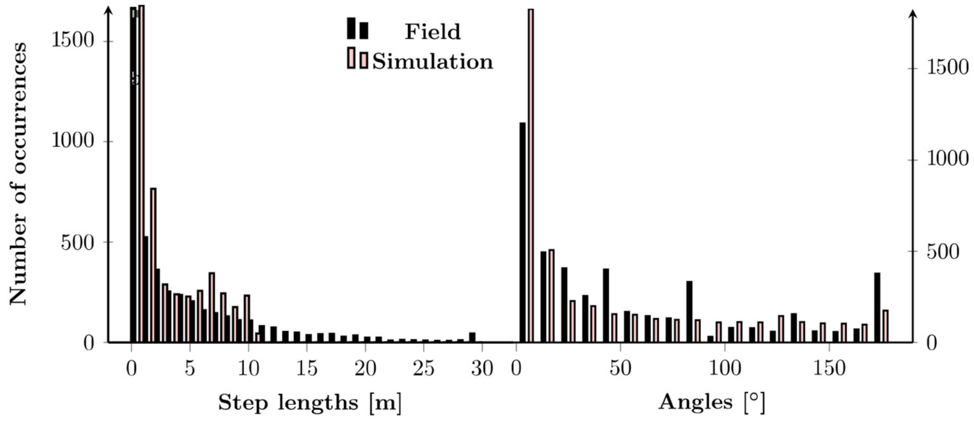

Figure S7. Histogram of step lengths (left) and angles (right) for field (black) and simulation (red) data during high food availability in plantations, with control parameters *abundanceReach* = 0.1, *explorationZone* = 2, *velocity* = 1 and *levyRatio* = 60.

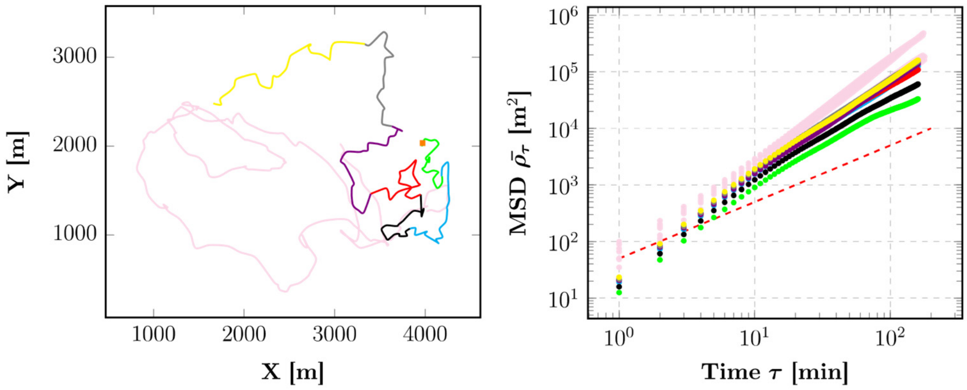

Figure S8. Evolution of the group position (left), with the corresponding MSDs (right, mean $\alpha = 1.56 \pm 0.09$) for a simulation of 7 consecutive days during high food availability in plantations, with control parameters *abundanceReach* = 0.1, *explorationZone* = 2, *velocity* = 1 and *levyRatio* = 60. Pink trajectories and MSDs represent the troop data. The orange square indicates the departure of the group of particles and the dashed line represents a line of a unitary slope, i.e., for $\alpha = 1$.

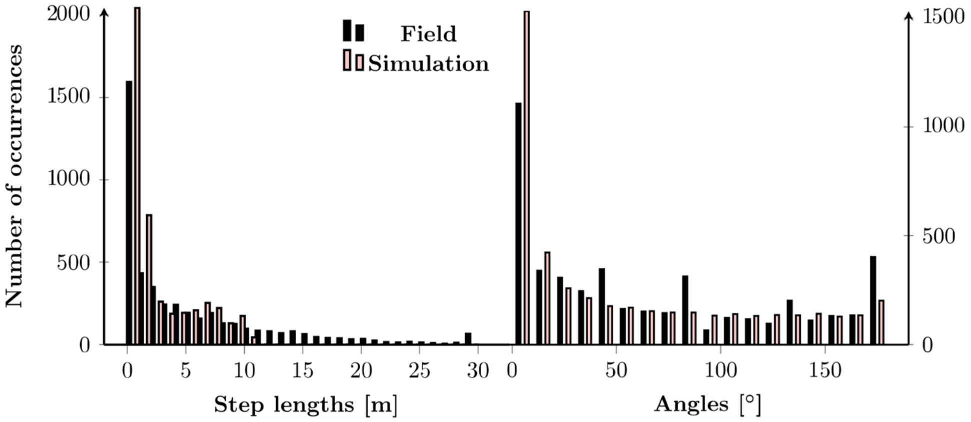

Figure S9. Histogram of step lengths (left) and angles (right) for field (black) and simulation (red) data during low food availability in dry evergreen forest and plantations, with control parameters *abundanceReach* = 0.1, *explorationZone* = 2, *velocity* = 1 and *levyRatio* = 35.

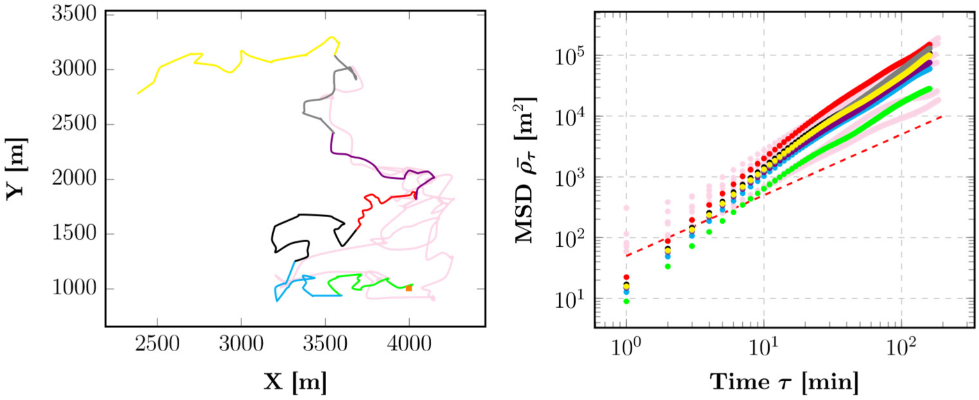

Figure S10. Evolution of the troop position (left), with the corresponding MSDs (right, mean $\alpha = 1.57 \pm 0.06$) for a simulation of 7 consecutive days during low food availability in dry evergreen forest and plantations, with control parameters *abundanceReach* = 0.1, *explorationZone* = 2, *velocity* = 1 and *levyRatio* = 35. Pink trajectories and MSDs represent the troop data. The orange square indicates the departure of the group of particles and the dashed line represents a line of a unitary slope, i.e., for $\alpha = 1$.

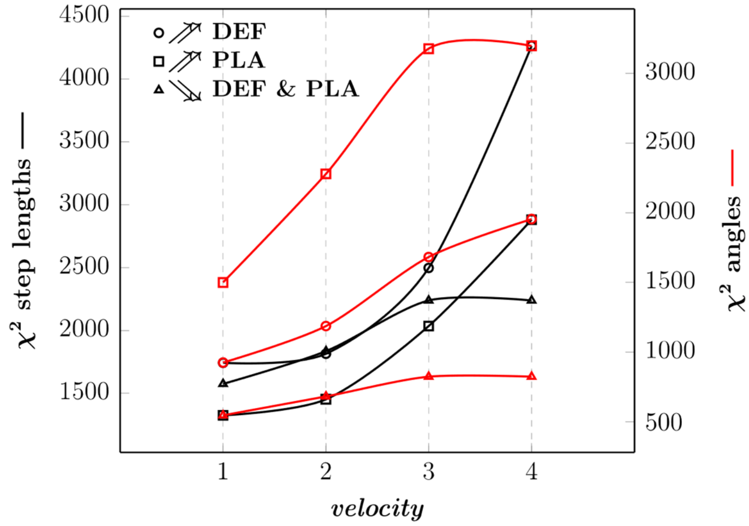

Figure S11. χ^2 for comparing step length and angle distributions between observed and simulated trajectories as a function of *velocity*, with control parameters *abundanceReach* = 0.1, *explorationZone* = 2 and *levyRatio* = 25. The black (resp. red) curves represent the χ^2 for step lengths (resp. angles) and are plotted along the left-hand (resp. right-hand) y-axis. For each period of food abundance, the group of particles was simulated from the same starting point.
